# Identifying favorable alleles for improving key agronomic traits in upland cotton

**DOI:** 10.1186/s12870-019-1725-y

**Published:** 2019-04-11

**Authors:** Panhong Dai, Yuchen Miao, Shoupu He, Zhaoe Pan, Yinhua Jia, Yingfan Cai, Junling Sun, Liru Wang, Baoyin Pang, Mi Wang, Xiongming Du

**Affiliations:** 1 State Key Laboratory of Cotton Biology, Institute of Cotton Research, Chinese Academy of Agricultural Sciences, Anyang, 455000 Henan China; 2 State Key Laboratory of Cotton Biology, Henan Key Laboratory of Plant Stress Biology, School of Life Sciences, Henan University, Kaifeng, 475000 China; 3 Agricultural College, Yangtze University, Jingzhou, 434000 China

**Keywords:** Upland cotton, Yield and fiber quality, Simple sequence repeats, Genome wide association study, Favorable alleles

## Abstract

**Background:**

*Gossypium hirsutum* L. is grown worldwide and is the largest source of natural fiber crop. We focus on exploring the favorable alleles (FAs) for upland cotton varieties improvement, and further understanding the history of accessions selection and acumination of favorable allele during breeding.

**Results:**

The genetic basis of phenotypic variation has been studied. But the accumulation of favorable alleles in cotton breeding history in unknown, and potential favorable alleles to enhance key agronomic traits in the future cotton varieties have not yet been identified. Therefore, 419 upland cotton accessions were screened, representing a diversity of phenotypic variations of 7362 *G. hirsutum*, and 15 major traits were investigated in 6 environments. These accessions were categorized into 3 periods (early, medium, and modern) according to breeding history. All accessions were divided into two major groups using 299 polymorphic microsatellite markers: G1 (high fiber yield and quality, late maturity) and G2 (low fiber yield and quality, early maturity). The proportion of G1 genotype gradually increased from early to modern breeding periods. Furthermore, 21 markers (71 alleles) were significantly associated (−log *P* > 4) with 15 agronomic traits in multiple environments. Seventeen alleles were identified as FAs; these alleles accumulated more in the modern period than in other periods, consistent with their phenotypic variation trends in breeding history. Our results demonstrate that the favorable alleles accumulated through breeding effects, especially for common favorable alleles. However, the potential elite accessions could be rapidly screened by rare favorable alleles.

**Conclusion:**

In our study, genetic variation and genome-wide associations for 419 upland cotton accessions were analyzed. Two favorable allele types were identified during three breeding periods, providing important information for yield/quality improvement of upland cotton germplasm.

**Electronic supplementary material:**

The online version of this article (10.1186/s12870-019-1725-y) contains supplementary material, which is available to authorized users.

## Background

As the leading natural fiber crop, cotton (*Gossypium* spp.) was grown on approximately 34.2 million ha with a total yield of approximately 2.62 × 10^7^ t in 2018, providing approximately 35% of the total fiber used worldwide [1–3]. China, India, and Pakistan consumed approximately 65% of the world’s raw cotton [4]. Upland cotton is native to Central America and was domesticated in the Yucatan peninsula approximately 5000 years ago. Of all the 4 cultivated cotton species, *G. hirsutum* shows the highest within-species phenotypic diversity [5, 6]. *G. hirsutum* has been bred for more than 150 years in China, source germplasms were introduced into China from the United States and the former Soviet Union prior to 1980 [7–9]. Until 2010, a total of 7362 cultivars had been collected in the National Mid-term Bank for Cotton in China [8]. To effectively explore these accessions, various efforts have been made to estimate genetic variation and candidate genes [10–12]. However, the core collection is also an effective way to access germplasm resources, which could alleviate the burden of managing germplasm collections. It can also simplify the process of screening exotic materials for plant breeders by reducing the size of surveyed materials [13, 14]. In most core collection studies, phenotype and genotype data have been used to measure genetic similarity [15]. In our previous study, a total of 419 upland cotton accessions had been chosen as the core collection from 7362 accessions [16, 17]. Recently, Ma et al. [18] also identified the traits-associated with SNPs and candidate genes of this core collection.

Association analysis is an alternative tool for testing quantitative trait loci (QTLs) and is a promising way to examine the anatomy of complex genetic traits in plants [11, 19–23]. Association analysis with simple sequence repeat markers (SSRs) has been widely used in previous studies of different crops, such as maize [24–26], rice [27, 28], soybean [29], oilseed rape [30] and cotton [31–34]. Frequently appearing alleles associated with important traits in elite accessions were defined as favorable alleles (FAs). To date, only a few SSR or SNP markers have been identified as FAs for complex traits in multi-environments [10, 12, 18]. In crops, FAs could be used to improve the target traits in subsequent marker-assisted selection breeding processes [35–38]. Analyzing the frequency and genetic effects of these alleles could improve our understanding of the origin and evolution of target traits. However, very few studies have examined the accumulation of FAs during multiple breeding stages in crops. Previously, several potential FAs for kernel size and milling quality were identified in wheat populations [39]. In cotton, only the frequency differentiation of FAs related to lint yield of 356 representative cultivars have been reported [36]. FAs related to fiber quality and favorable allele accumulation conditions in multiple breeding periods are still unknown.

In the present study, a total of 419 upland cotton accessions [16, 17] and 299 SSR markers were used to perform a genome-wide association study (GWAS) and examine genotype proportions during three breeding periods. Additionally, we identified accumulation conditions of FAs in all accessions and discuss their effects on fiber yield and quality in cotton cultivars in different breeding periods. Results of this study will provide an effective way to identify potentially useful FAs and accessions for improving fiber quality and yield.

## Methods

### Plant materials

**Fig. 1:**
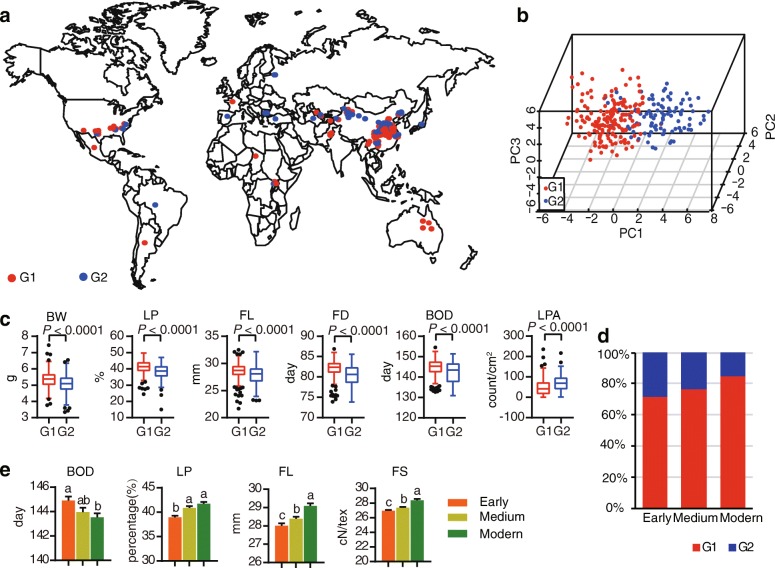
Geographic distribution and population variation of upland cotton accessions. **a** The geographic distribution of upland cotton accessions. Each dot of a given color on the world map represents the geographic distribution of the corresponding cotton accession groups. **b** Principal component analysis (PCA) plots of the first two components for all accessions. **c** Variance analysis of six phenotype traits between two groups, with black points representing mild outliers. In box plots, center line indicates median; box limits indicate upper and lower quartiles; whiskers denote 1.5× interquartile range; points shows outliers. BW: boll weight; LP: lint percent; FL: fiber upper half mean length; FD: flowering date; BOD: boll opening date; LPA: leaf pubescence amount.  *P* values in this and all other figures were derived with in Duncan’s multiple comparison tests. **d** Percentages are shown in a stacked column chart for 3 breeding periods (early, medium, and modern). **e** Four traits are compared among three breeding periods. **a**, **b**, **c** above the bars show significant differents (*P* < 0.05)

We sampled 419 *Gossypium hirsutum* accessions [16, 17] that were assembled for genotyping and phenotyping. The accessions were derived from 17 diverse geographic origins, including China, the United States, the former Soviet Union, Australia, Brazil, Pakistan, Mexico, Chad, Uganda, and Sudan, which are the main cotton-growing areas throughout the world (Fig. 1a, Additional file 1: Table S2). All accessions, which were introduced or bred from 1918 to 2012, were divided into 3 breeding periods: 1920s to 1980s (early, *n* = 151), 1980s to 2000s (medium, *n* = 157), 2001s to 2012s (modern, *n* = 111) (Additional file 1: Table S2). The accessions were authorized for use by the Cotton Research Institute, Chinese Academy of Agriculture Sciences, Anyang, Henan Province (Additional file 1: Table S2).

### Phenotypic design and statistical analysis

A 6-environment experiment was designed for phenotyping at 3 different locations in 2014 and 2015. The 3 locations were Anyang (AY) in Henan Province, Jingzhou (JZ) in Hubei Province, and Dunhuang (DH). A total of 15 agronomic traits were investigated, including maturity, trichome, yield, and fiber quality. All traits were scored in six environments except stem pubescence amount (SPA) in 2014 and leaf pubescence amount (LPA, count/cm^2^) in 2015 [17, 18]. Sympodial brand number (SBN) was counted after topping. Flowering date (FD, day) was calculated as the days from the sowing day to the day that half of the plants had at least one open flower for each environment. Boll open date (BOD, day) was the number of days from the sowing day to the day that half of the plants had at least one boll open in one accession in each environment. Thirty naturally mature bolls from each accession were hand-harvested to calculate weight per boll (BW, g) and gin fiber. Seed index (SI, g) was the weight of 100 cotton seeds. Fiber samples were separately weighed for calculating lint percentage (LP, %), fiber yellowness (FY), fiber upper half mean length (FL, mm), fiber strength (FS, cN/tex), fiber elongation (FE, %), fiber reflectance rate (FRR, %), fiber length uniformity (FLU), and spinning consistency index (SCI). Previously, an ANOVA was performed to evaluate the effects of multiple environments (Additional file 2: Table S5) [17, 18]. Best Linear Unbiased Prediction (BLUP) [18, 40] was used to estimate phenotypic traits across 6 environments based on a linear model. Averages of three replicates within the same environment for each accession were used when analyzing phenotypic data. All statistical analyses were calculated using SAS9.21 software.

### Molecular marker genotyping

Each young leaf tissue sample was collected from a single plant and DNA was extracted using the procedure described by Li et al. [41] and Tyagi et al. [42]. To identify polymorphic SSR markers in 419 upland cotton accessions, in this study, twenty-four diversity accessions (Additional file 1: Table S2 in black) were used as a panel to screen 1743 polymorphism markers from 5000 SSR markers, finally all 419 accessions were used to screen 299 polymorphism markers from 1743 SSR markers. Information on these SSR microsatellite markers are available in CottonGen (http://www.cottongen.org) (Additional file 3: Table S3). We used ‘0’ as no band and ‘1’ as a band. The combinations of ‘0’ and ‘1’ represented alleles of each marker.

### Population structure and LD analysis

Three methods were used to estimate the number of subgroups in the cotton accessions based on the genotypic database. First, the number of simulation subgroups (K value) was set from 1 to 12. The natural logarithms of probability data (LnP(K)) and ΔK were calculated using MS Excel 2016. ΔK was set as the primary factor for estimating the excellent value of K [43]. STRUCTURE 2.3.4 software [44] was used to calculate Bayesian clustering from K = 1 to 12 for 5 repetitions. Second, the genotypic principle component analysis (PCA) provided the top 3 eigen-vectors, PC1, PC2, and PC3, using R (https://cran.r-project.org/). Third, power marker 3.25 was used to calculate the genetic distance among accessions using a neighbor-joining (NJ) phylogeny based on Nei’s genetic distances [45, 46].

### Association analysis

Marker-trait association analyses for 15 agronomic traits in 6 environments were conducted using a mixed linear model with the TASSEL 2.0 software [11, 32, 47, 48]. The MLM-incorporated kinship (K-matrix) was corrected for both Q-matrix and K-matrix (MLM (Q + K)) to reduce errors from population structure. The threshold for the significance of associations between SSR markers and traits was set as *P* < 0.0001 (−log *P* > 4). The sequences of significantly associated markers were searched from CottonGen Database (http://www.cottongen.org) and assigned a genome location (NAU-genome database of TM-1, Zhang et al., 2015) [49]. The allele effect for phenotype was estimated as follow method [39, 50]:

$$ {\mathrm{a}}_{\mathrm{i}}=\sum {\mathrm{x}}_{\mathrm{i}\mathrm{j}}/{\mathrm{n}}_{\mathrm{i}}-\sum {\mathrm{N}}_{\mathrm{k}}/{\mathrm{n}}_{\mathrm{k}} $$

where a_i_ was the phenotype effect of I allele, x_ij_ was the phenotype value of j individual with i allele, n_i_ was the total individuals with i allele, N_k_ was the phenotype value of j individual with null i allele and n_k_ was the total individuals with null i allele.

### Favorable alleles (FAs) identification

In our study, the favorable alleles (FAs) indicated the alleles which were benefited for cotton traits improvement. Their definition was described as follow:

For each trait, according to the GWAS result, the corresponding phenotypical data of the locus (SSR marker) with the largest -log *P* value was used to compare the genetic effect between alleles. The allele with larger trait value (except maturity) were defined as favorable allele (FA).

## Results

### Geographic distribution and genetic and phenotypic features of the upland cotton core collection

A total of 419 accessions were collected from 17 countries (Fig. 1a, Additional file 1: Table S2), including 319 from China, 55 from the United States, and 16 from the former Soviet Union. A total of 299 polymorphic markers (1063 alleles) were selected, covering the 26 chromosomes in upland cotton (Additional file 4: Figure S1). A summary of these markers and their polymorphisms is provided in Additional file 5: Table S1. A total of 419 upland cotton accessions were analyzed using the 299 SSR markers. The polymorphism information content (PIC) value of each marker ranged from 0.002 to 0.85, with an average of 0.54 (Additional file 3: Table S3). The average PIC of Ne and H′ was 2.47 and 0.91, respectively (Additional file 5: Table S1, Additional file 3: Table S3, Additional file 4: Figure S1). Among the markers, chromosome 5 had the largest number of markers (19), while chromosome 13 had the least (4). On average, 11.4 markers were distributed on each chromosome and 3.5 alleles (range: 2–7) were generated per SSR marker.

The LD decay distance was determined by calculating pairwise correlation coefficient (R^2^) decay from its maximum value (0.47 kb) to its half value at 304.8 kb for the whole population (Additional file 6: Figure S2). The LD decay distance in this study was slightly higher than what was reported by Wang et al. (296 kb) [12], but lower than decay distances reported by Ma et al. (742.7 kb) [18] and Fang et al. (1000 kb) [10].

Two clusters were identified in the core collection based on ΔK value (Additional file 7: Figure S3). A neighbor-joining tree was constructed based on Nei’s genetic distances [46], and the two major clusters were defined as G1 (322 accessions) and G2 (97 accessions) (Fig. 1b, Additional file 1: Table S2). Genetic relationships among accessions were further studied using principal component analysis (PCA) (Fig. 1b). The two major groups were also well separated by plotting the first three components (PC1 to PC3). Overall, the results of the STRUCTURE, PCA, and phylogeny tree consistently confirmed that two sub-groups exist in the upland core collection based on SSR markers (Fig. 1b, Additional file 7: Figure S3).

For phenotypic core collection data, a wide range of phenotypic variation was observed when 15 agronomic traits were investigated in six environments. The coefficients of variation (CV) for leaf pubescence amount (LPA) was > 60%, and the CVs in stem pubescence amount (SPA) and seed index (SI) were > 10%. Boll weight (BW), lint percentage (LP), and spinning consistency index (SCI) CVs were approximately 10%. The CVs for fiber elongation (FE), fiber length uniformity (FLU), fiber reflectance rate (FRR), and flowering date (FD) were < 5% and CVs of other traits ranged from 5 to 10% (Additional file 8: Table S4). Additionally, Pearson’s correlation coefficient was estimated for all investigated traits and results show a negative correlation between LPA and FD (FD and BOD) and a positive correlation between growth period and fiber yield/fiber quality traits (Additional file 9: Figure S4). Most yield- and fiber quality-related traits of G1 were significantly higher than G2 except SPA, LPA, and SI (Fig. 1c, Additional file 10: Figure S5a). Further comparisons of accessions among the three breeding periods showed that the G1 genotype proportion gradually increased over time (Fig. 1d) and G2 was shown the opposite trend. In this study, we found that most yield- and fiber quality-related traits significantly increased with three breeding periods (Fig. 1e, Additional file 10: Figure S5b). This finding is consistent with the cotton breeding targets (fiber quality and yield improvement) over the past fifty years.

### Identification of trait-associated alleles by GWAS

The association analysis was based on best linear unbiased prediction (BLUP) traits and 299 SSR markers across six environments in 419 upland cotton accessions. Significantly associated SSR markers were detected for all the traits using a mixed linear model (MLM) at -log *P* > 4 (Table 1). We mapped 278 SSR marker loci onto 26 upland cotton chromosomes (Additional file 11: Figure S6), a total of 21 markers (73 alleles) were determined to have significant associations with 15 traits, including 7 fiber quality traits (FS, FL, FRR, SCI, FE, FLU, and FY), 3 yield-related traits (BW, LP, and SI), 2 trichomes-related traits (LPA and SPA) and 3 maturity traits (FD, BOD, and SBN). Thirteen of these markers were detected in at least 2 environments and 12 were pleiotropic markers that were associated with more than one trait (Table 1).

**Table 1:** Associations analysis detected among 15 agronomic-related traits

Type	Traits	Loci^A^	Physical chromosome	Physical position (Mb)	Effect for phenotype	-log *P*^B^	Known loci^C^
Fiber quality	FL	**BNL3452**	A05	3.86	0.39	4.51(d)	[59, 60]
		**CM0043**	A08	10.80	1.64/1.79/1.64/13.22/14.28/0.09/1.31/1.53	5.80(j)/6.02(c)/5.86(f)/4.09(i)/4.60(a)/4.03(d)/5.21(b)/6.62(e)	
		**NBRI_GE21415**	D01	28.06	1.31/0.23/− 2.13/14.17/14.09	4.41(j)/5.01(c)/4.64(b)/4.13(e)/4.01(h)	[61]
	FS	BNL2960	A10	91.23	−1.11/− 1.25	4.16(i)/4.02(h)	
		**CM0043**	A08	10.80	18.87/1.07/1.85	4.23(j)/4.92(e)/4.01(f)	
		**NAU3201**	D08	56.55	−1.15/−2.13	5.23(c)/4.14(b)	
		**NBRI_GE21415**	D01	28.06	1.34	4.19(b)	[61]
	FE	**HAU2631**	A13	2.16	3.81/3.02	4.79(c)/4.49(a)	[55, 62]
		**NAU3084**	A12	86.65	0.15	4.1(a)	
		**NBRI_GE21415**	D01	28.06	1.25	4.41(e)	[61]
	FY	NAU2914	D08	39.43	−0.26	4.05(i)	
		**NAU3291**	D12	48.92	0.95/0.83	4.77(i)/4.25(j)	
	FLU	**CM0043**	A08	10.80	13.25/1.53	4.63(j)/5.95(c)	
		**HAU2631**	A13	2.16	3.51/3.96/1.55/0.24/4.51	4.78(j)/4.27(f)/5.18(i)/5.72(g)/4.20(e)	
		NAU2980	D13	59.74	−2.14/−0.66/−0.14	4.30(j)/4.07(c)/4.91(h)	
		**NBRI_GE21415**	D01	28.06	4.46/2.20/0.47/1.33/−1.08	5.41(c)/4.78(i)/6.16(g)/4.42(h)/5.34(j)	[61]
	SCI	**BNL3452**	A05	3.86	8.10	4.06(d)	
		**CM0043**	A08	10.80	1.58/0.95/14.53/13.44/16.92	5.08(j)/5.00(c)/4.92(f)/4.63(b)/4.75(e)	
		**NBRI_GE21415**	D01	28.06	0.17/0.18/1.78/0.14/1.06/0.14/0.64/8.95	5.23(j)/4.91(c)/4.29(f)/4.97(i)/4.25(d)/5.02(g)/5.72(b)/4.10(e)	
	FRR	**NAU3291**	D12	48.92	0.92/0.70	4.72(j)/4.88(i)	
Yield	BW	BNL3867	D12	12.51	2.18	5.06(d)	
		NAU3587	A08	0.40	−0.27	4.06(i)	
		**NBRI_GE21415**	D01	28.06	13.00/1.64/1.18	4.03(j)/4.82(c)/4.59(e)	[61]
	LP	**HAU2631**	A13	2.16	3.87/4.52/3.51/0.14/0.52/1.15/1.86/4.36	4.95(j)/4.29(c)/4.46(f)/5.55(i)/6.08(g)/5.47(b)/5.16(e)/4.66(h)	
		HAU3073	D11	17.05	0.26	4.21(h)	
	SI	**CM0043**	A08	10.80	1.18	5.19(a)	
	SBN	BNL3408	A03	2.82	−0.51	4.25(b)	
		**NAU3084**	A12	86.65	−1.29	4.04(b)	
		NAU3434	D10	12.45	0.32	6.64(d)	
Maturity	BOD	**JESPR0190**	D10	60.14	−2.5/−10.59/3.24	4.43(j)/5.24(f)/4.50(b)	
		**NAU3201**	D08	56.55	−1.76	5.18(a)	
		**NBRI_GE10433**	A06	92.58	2.34	4.21(i)	
	FD	**JESPR0190**	D10	60.14	−8.01/−3.93	5.62(f)/8.06(d)	
		**NAU0874**	A06	93.20	2.40	4.62(d)	[51, 52, 55]
		**NAU5433**	A06	89.92	0.20	4.4(d)	[51, 52, 55]
Trichome	LPA	**NAU0874**	A06	93.20	0.18/−2.53	13.84(b)/10.49(h)	[52, 55]
		**NAU5433**	A06	89.92	−2.31/2.61	9.63(b)/9.92(h)	[51, 52, 55]
		**NBRI_GE10433**	A06	92.58	−3.04/−1.59	13.23(b)/10.56(h)	
		**NBRI_GE18910**	A06	93.75	−2.20/−0.18	7.99(b)/6.21(h)	
	SPA	**NAU0874**	A06	93.20	4.86/−1.97/−5.86	4.39(i)/12.59(a)/5.06(h)	[52, 55]
		NAU5164	D12	40.11	0.24/0.19	4.16(g)/4.70(i)	
		**NAU5433**	A06	89.92	0.27/−1.72/4.58	4.84(a)/9.06(h)/5.41(i)	[51, 52, 55]
		**NBRI_GE10433**	A06	92.58	−1.77/−5.43	13.09(a)/4.35(h)	
		**NBRI_GE18910**	A06	93.75	−4.47/−0.44	6.93(a)/4.95(h)	

Common markers detected in more than two traits are shown bold in **A** and the negative logarithm value of *P* of each associated marker is shown in **B**. Different environments: represented the environment of Anyang in 2014, Anyang in 2015, the BLUP value of Anyang in 2014 and 2015, Dunhuang in 2014, Dunhuang in 2015, the BLUP value of Dunhuang in 2014 and 2015, Jingzhou in 2014, Jingzhou in 2015, the BLUP value of Jingzhou in 2014 and 2015, the BLUP value of 6 environments, respectively. **C** shows a comparison between trait-marker associations identified in this study and QTLs identified in previous studies

In 7 fiber-associated markers, CM0043 was found to be associated with 1 yield-related and 4 fiber quality traits (LU, SI, SCI, FS, and FL), with the strongest association for FL (−log *P* = 6.02). This marker has been reported to be linked with a major fiber strength QTL in two other population studies (Cai et al. 2014a; Kumar et al. 2012). HAU2631 was associated with 1 yield- and 2 fiber quality-related traits, including FE, FLU, and LP, and was located on the confidence interval of a previously identified FE QTL (Tang et al. 2015). A total of 6 markers were associated with the other 4 traits (BOD, FD, LPA, and SPA). Among these markers, NBRI_GE18910 was associated with trichomes (LPA and SPA), JESPR0190 was associated with maturity (FD and BOD), and the pleiotropic markers NAU5433 and NAU0874 were both associated with maturity- and trichome-related traits (LPA, SPA, and FD). Previously, these 2 markers (NAU5433 and NAU0874) were thought to be located on a cotton trichome locus (*T1*) [51, 52]. Our study is the first to reveal the pleiotropic effect of this locus and show the possible relationship between maturity and trichome in cotton.

### Accumulation of FAs for important traits in three cotton breeding periods

**Fig. 2:**
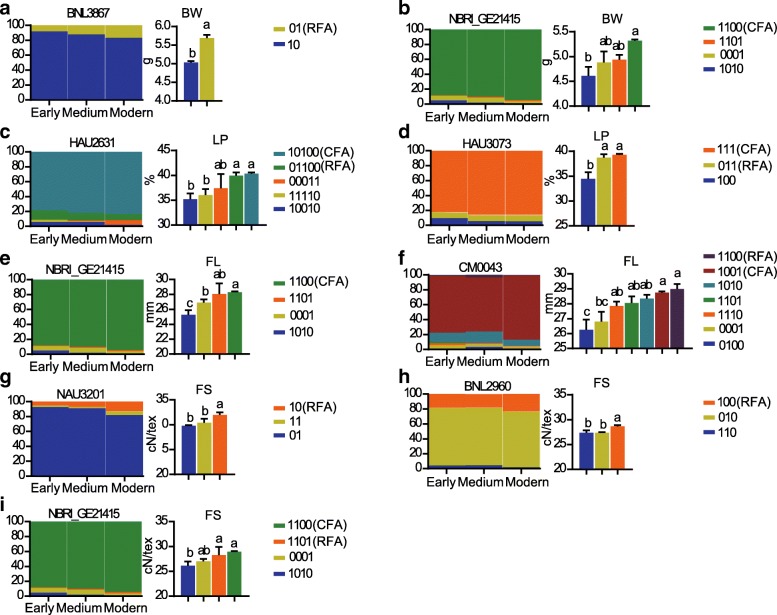
The distribution and genotyping of favorable alleles related fiber-yield and quality traits among three breeding periods in 419 upland cotton accessions. The distribution and genotyping of the alleles of BNL3867, NBRI_GE21415, HAU2631, HAU3073, NBRI_GE21415, CM0043, NAU3201, BNL2960, NBRI_GE21415 locus was shown in **a-i** (left chart). **a-i** (left chart) Frequency pile-up diagram for different genotypes among three breeding periods (early, medium, and modern varieties). Histogram for genotyping different traits was shown in the right chart). BW: weight per boll, LP: lint percentage, FL: fiber upper half mean length, FS: fiber strength. RFA indicated rare favorable alleles with the frequency of FAs < 25% and CFA was common favorable alleles with FAs > 70%. *P* values in this and all other figures were derived with in Duncan's multiple comparison tests. The letters **a**, **b**, **c** above the bars show significant differents (*P *< 0.05)

We identified FAs, which were alleles associated with significantly better traits (higher yield/fiber quality and shorter maturity period), by analyzing phenotype and allele frequency data for each marker in 3 breeding period populations. A total of 21 markers (carried30 FAs) that were associated with yield- fiber quality-related traits and maturity traits (BOD, FD) exhibited a clearly selective trend that corresponded to human demands during the 3 breeding periods (Fig. 2, Additional file 12: Figure S7). In these markers, the frequency of FAs significantly increased with the breeding period. This finding was similar results from our previous SNP-based study [18]. However, 15 alleles were found to be lost in the modern population, such as NBRI_GE21415_1010 for BW, HAU2631_11110 for LP, NBRI_GE21415_1010 and CM0043_1101 for FL, and NBRI_GE21415_1010 for FS (Fig. 2, Additional file 12: Figure S7). This result showed that the level of genetic diversity in the whole population was decreasing along with the intentional selection of FAs by humans during breeding progress. Moreover, 2 typical frequency distributions occurred for FAs in all accessions (Fig. 2, Additional file 12: Figure S7). The FAs for each marker were further categorized as common FA (CFA) or rare FA (RFA). A total of 13 CFAs and 17 RFAs and were identified (Fig. 2, Additional file 12: Figure S7) which associated with yield- fiber quality-related traits and maturity traits (BOD, FD). For example, HAU2631_10100 was a CFA for LP and BNL3867_01 was an RFA for boll weight. CFAs are commonly selected in early breeding stages due to their widespread existence in most of the accessions, while RFAs might appear in later stages and have greater potential for future breeding utilization.

### The contribution and potential of FAs in 419 upland cotton accessions

**Fig. 3:**
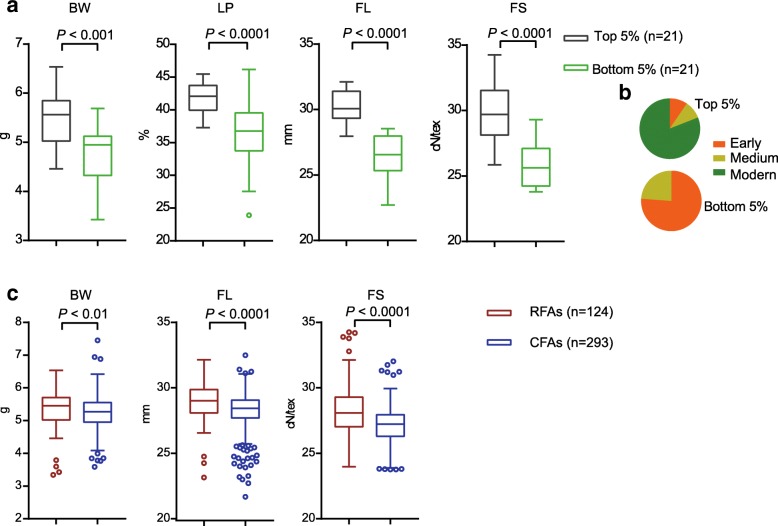
Phenotypic characteristics of accessions containing FAs, CFAs and RFAs. **a** Yield and fiber quality characteristics of accessions with more FAs (top 5%) and fewer FAs (bottom 5%), respectively. BW: weight per boll, LP: lint percentage, FL: fiber upper half mean length, FS: fiber strength. **b** The proportion of accessions with more FAs (top 5%) and fewer FAs (bottom 5%) in 3 periods (orange Early, golden Medium, green Modern), respectively. **c** Yield and fiber quality characteristics of accessions containing CFAs and RFAs, respectively. Horizontal lines in the box plots represent the minimum, lower quartile, median, upper quartile, and maximum, respectively, and blue and red points represent mild outliers. In box plots, center line indicates median; box limits indicate upper and lower quartiles; whiskers denote 1.5× interquartile range; points shows outliers. *P* values in this and all other figures were derived with Duncan's multiple comparison tests

To evaluate the contribution of FAs in 419 upland cotton accessions, we calculated the total number of FAs in each accession (Additional file 13: Table S6, Fig. 3), sorted by count order, and analyzed the major traits of the top and bottom 5% accessions (Additional file 14: Table S7, Fig. 3a-b). For both fiber yield- and quality-related traits, the accessions carried more FAs (top 5%) were significantly higher than those carried fewer FAs (bottom 5%) (Fig. 3a-b). We also found that most of the top 5% accessions were developed in modern and medium breeding periods, but all the bottom 5% accessions were developed in early and medium breeding periods (Fig. 3b). This result highlights the large contribution of FAs for cotton germplasm improvement during breeding progress. We also studied the effects of CFAs and RFAs, and accessions that contained more than 1 RFA were categorized to compare with non-RFA accessions (Fig. 3c, Additional file 15: Table S8,). In maturity- and fiber quality-related traits, RFAs showed a significantly greater effect than CFAs despite the small proportion of RFAs in the population (Fig. 3c). This result suggests that both maturity and fiber quality may have more potential for improvement by utilizing RFAs in the future.

## Discussion

### Identification of new trait-associated and pleiotropic SSR markers by using 419 upland cotton accessions

Previously, several SSR markers were determined to be associated with agronomic traits using molecular markers [34, 53, 54]. In our study, we identified 21 SSR markers significantly associated with key agronomic traits by using a large and diverse panel of upland cotton core collection with clear genetic backgrounds and multi-environmental data. Sixteen new markers associated with key traits were reported (Table 1). For example, NBRI_GE10433 located on chromosome A06 was associated with maturity and trichome, and CM0043 located on chromosome A08 was associated with yield and fiber quality (Table 1). Importantly, we found new pleiotropic SSR markers enriched in specific chromosomal regions on the genome. These regions may harbor causal genes which underlie the genetic basis for important traits in cotton. Four markers (NAU0874, NAU5433, NBRI_GE10433, and NBRI_GE18910) were enriched in a 3.3 Mb-length range at the end of chromosome A06. These markers were found to be associated with maturity- (FD, BOD) and trichome-related traits (LPA, SPA). Previously, only NAU0874 and NAU5433 were reported to be linked with *T1*, a locus controlled by trichome traits in both *G. barbadense* [51] and *G. hirsutum* [52]. Our study was the first to reveal that this locus might be also associated with maturity. Interestingly, the region next to the *T1* locus was also suggested to be associated with fiber yield (LP) and fiber quality traits (FL, FU, FM, FS) in fine mapping studies [55, 56]. Therefore, genes in this region may play an important role in pleiotropically regulating cotton phenotypes, though further research is needed.

### RFAs as potential molecular markers for future upland cotton fiber quality improvement

Recently, several microarrays- and SNP-based studies reported a large set of SNP markers associated with various traits in upland cotton [11, 18, 57] However, due to the lack of genetic diversity and pedigree information, population structure characteristics were still not clear, making it difficult to genetically distinguish the accessions according to breeding periods. A recent study demonstrated that upland cotton developed in different periods could be divided by molecular markers when choosing representative accessions [58]. Therefore, material panel selection was the key factor for identifying period-specific or FAs. In this study, we comprehensively considered phenotypic and genetic variations, genetic background, geographical distribution, and recorded pedigree when choosing materials [16, 17], and found some strong associated rare favorable alleles for potential improvement of fiber yield, fiber quality, maturity, etc. Based on SSR markers, the whole panel could be genetically divided into two sub-groups: G1 (higher fiber yield and quality but later maturity) and G2 (lower fiber yield and quality but earlier maturity) (Fig. 1). Comparisons of genetic and phenotypic variation between the 2 sub-groups indicated that the G1 genotype proportion gradually increased from early to modern periods (Fig. 1), which showed that fiber yield and fiber quality FAs accumulated with time (Fig. 3). Additionally, within FAs, the RFAs had a greater effect than CFAs for fiber quality traits, showing their potential for fiber quality improvement in upland cotton (Fig. 3). In breeding practice, fiber quality (fiber length and strength) was commonly negatively correlated with fiber yield (boll weight), especially for superior fiber quality accessions. For example, Suyou 610 (FL: mean = 32.1 mm, FS: mean = 33.8 cN/tex, BW: mean = 4.5 g) and J02508 (FL: mean = 32.1 mm, FS: mean = 33.9 cN/tex, BW: mean = 4.4 g) (Additional file 16: Table S9) were superior fiber quality accessions that contained more RFAs than other accessions. Moreover, as fiber quality/yield was negatively correlated with early maturity in cotton, most early maturity accessions that contained RFAs had low fiber/yield quality. Results from this study suggest that RFAs accumulated in a few accessions may produce super traits (strongest fiber/yield quality or earliest maturity). Thus, more RFAs should be considered to utilize in the future. For example, potential accessions speedily by identified RFAs such as Xinluzhong 34 (FL: mean = 29.6 mm, FS: mean = 29.1 cN/tex, LP: mean = 45.5%, FD = 83.0 d, BOD = 147.3 d), Xinluzhong 5 (FL: mean = 31.9 mm, FS: mean = 34.3 cN/tex, BW: mean = 4.0 g, FD = 78.0 d, BOD = 144.7 d), Kuche 96,515 (FL: mean = 30.0 mm, FS: mean = 29.4 cN/tex, FD = 76.0 d, BOD = 143.9 d), and Caike 510 (FL: mean = 30.8 mm, FS: mean = 30.4 cN/tex, BW: mean = 6.3 g, FD = 81.7 d, BOD = 145 d) had suitable maturity and higher fiber/yield quality (Additional file 16: Table S9). These results provide a new understanding of the genetic variation and accumulation of FAs in upland cotton breeding history. Further, we identified several RFAs and potential accessions by screening molecular markers to improve genetic resources and cotton breeding.

## Conclusion

The 419 upland cotton accessions were collected from 17 countries, which genotyped using 299 SSR markers and clustered into two sub-groups (G1, G2) var. G1 (high fiber yield and quality, late maturity) and G2 (low fiber yield and quality, early maturity). G1 and G2 were correlated with 3 breeding periods. The proportion of G1 genotype gradually increased from early to modern breeding periods. Twenty-one SSR markers (73 alleles) were identified and associated with 15 agronomic traits. Identification of new trait-associated and pleiotropic SSR markers by using 419 upland cotton accessions. Two types of FAs (13 CFAs and 17 RFAs) were identified FAs were accumulated during 3 breeding periods, especially for CFAs. The potential elite accessions could be rapidly identified by RFAs. This study provides a new understanding of genetic variation and FAs accumulation in upland cotton breeding history and shows that the screening of molecular markers could accelerate genetic resources enhancement and breeding in upland cotton.

## Additional files

Additional file 1: **Table S2.** List of 419 upland cotton accessions was used in this study and their cluster information. (XLSX 44 kb)

Additional file 2: **Table S5.** Mean squares of the ANOVA of 15 agronomic traits measurements in 6 environments. “*”, “**” indicate significance at the probability levels of *P* < 0.01 and *P* < 0.001, respectively. (XLSX 10 kb)

Additional file 3: **Table S3.** List of 299 SSR markers was used in this study and their position information and genetic diversity. (XLSX 46 kb)

Additional file 4: **Figure S1.** The diversity of 299 SSR markers. **a** Diversity index, **b** polymorphism information content, **c** distribution of markers in the upland cotton genome. (PDF 194 kb)

Additional file 5: **Table S1.** Summary of 299 SSR polymorphisms. (XLSX 9 kb)

Additional file 6: **Figure S2.** Linkage disequilibrium decay (R^2^). (PDF 222 kb)

Additional file 7: **Figure S3.** Population structure of the 419 accessions. **a** Mean LnP(D) values plotted from 1 to 12, **b** Ln(D) values plotted from 1 to 12, **c** Population structure of the 419 accessions based on STRUCTURE when K = 2, and **d** Neighbor-joining three of all accessions constructed from whole-genome SSRs. (PDF 433 kb)

Additional file 8: **Table S4.** Summary statistics of 15 agronomic traits in 419 upland cotton accessions. (XLSX 14 kb)

Additional file 9: **Figure S4.** Frequency distribution of phenotypic variation of 15 agronomic traits and correlation coefficients among traits in 419 accessions. Abbreviations are defined as (SPA) stem pubescence amount, (LPA) leaf pubescence amount, (BOD) boll open date, (BW) weight per boll, (FE) fiber elongation, (FD) flowering date, (FS) fiber strength, (LP) lint percentage, (FLU) fiber length uniformity, (FRR) fiber reflectance rate, (SBN) sympodial brand number, (SCI) spinning consistency index, (SI) seed index, (FL) fiber upper half mean length, and (FY) fiber yellowness. (PDF 2459 kb)

Additional file 10: **Figure S5.** The variance analysis of phenotype traits in 2 groups and 3 breeding periods. **a** Comparison of investigated traits between 2 groups. **b** Comparison among 3 breeding periods. Horizontal lines in the boxplot represent the minimum, lower quartile, median, upper quartile and maximum, respectively, and black points represent mild outliers. *P* values in this and all other figures were derived within Duncan’s multiple comparison tests. Abbreviations are defined as (BW) weight per boll, (FD) flowering date, (FE) fiber elongation, (FS) fiber strength, (FLU) fiber length uniformity, (SBN) sympodial brand number, (FY) fiber yellowness, (LPA) leaf pubescence amount, (FRR) fiber reflectance rate, (SCI) spinning consistency index, (SI) seed index, and (SPA) stem pubescence amount. (PDF 316 kb)

Additional file 11: **Figure S6.** Physical map of 278 markers associated with 15 agronomic traits in 419 accessions. The loci with lines below the name were the markers significant associated (−log *P* > 4) with agronomic traits. Map distances were given in megabyte (Mb). The phenotypes of GWAS were within parentheses. Phenotypes were shown as stem pubescence amount (SPA), leaf pubescence amount (LPA), boll open date (BOD), weight per boll (BW), fiber elongation (FE), flowering date (FD), fiber strength (FS), lint percentage (LP), fiber length uniformity (FLU), fiber reflectance rate (FRR), sympodial brand number (SBN), spinning consistency index (SCI), seed index (SI), fiber upper half mean length (FL), and fiber yellowness (FY). (PDF 1224 kb)

Additional file 12: **Figure S7.** Allele distribution of 11 fiber-yield and quality traits and favorable alleles found in 419 upland cotton accessions early, medium, and modern varieties. **a-k** Frequency pile-up diagram for alleles distribution of 4 yield-fiber quality loci in 419 upland cotton accessions early, medium, and modern varieties (left). Histogram for 11 yield-fiber quality traits, based on the alleles of different loci, respectively (right). The significance (*P* < 0.05) was tested by Duncan’s multiple comparison tests. We also identified rare favorable alleles (RFAs) as those that were the frequency of FAs < 25% and common favorable alleles (CFAs) as the frequency of favorable alleles > 70%, respectively. **a-k** Different traits in early, medium, and modern periods, **a** (BOD) boll open date, **b** (FD) flowering date, **c** (LPA) leaf pubescence amount, **d** (SPA) stem pubescence amount, **e** (FE) fiber elongation, **f** (FLU) fiber length uniformity, **g** (FY) fiber yellowness, **h** (SCI) spinning consistency index, **i** (FRR) fiber reflectance rate, **j** (SI) seed index, and **k** (SBN) sympodial brand number. (PDF 724 kb)

Additional file 13: **Table S6.** The frequency of favorable alleles in 3 breeding periods. (XLSX 14 kb)

Additional file 14: **Table S7.** Favorable alleles for the top 5% and bottom 5% accessions in 4 yield and fiber quality traits. (XLSX 11 kb)

Additional file 15: **Table S8.** Categorization of accessions containing more than one rare favorable allele (RFA) and non-RFA accessions. (XLSX 25 kb)

Additional file 16: **Table S9.** Summary of 2 types of favorable alleles in key agronomic traits. (XLSX 9 kb)

## Abbreviations

BOD: Boll opening date
BW: Boll weight
FD: Flowering date
FE: Fiber elongation
FL: Fiber upper half mean length
FLU: Fiber length uniformity
FRR: Fiber reflectance rate
FS: Fiber strength
FY: Fiber yellowness
RFA: Rare favorable allele
GWAS: Genome-wide association studies
LD: Linkage disequilibrium
LP: Lint percent
LPA: Leaf pubescence amount
MLM: Mixed linear model
PCA: Principal component analysis
PFA: Potential favorable allele
QTL: Quantitative trait loci
SBN: Sympodial brand number
SCI: Spinning consistency index
SI: Seed index
SPA: Stem pubescence amount
SSR: Sequence repeat marker
TASSEL: Trait Analysis by Association Evolution and Linkage
