# Supplementary figures and images for: Identifying favorable alleles for improving key agronomic traits in upland cotton
Source: BMC Plant Biol. 2019 Apr 11;19:138. doi: 10.1186/s12870-019-1725-y (PMC6458685; doi:10.1186/s12870-019-1725-y)

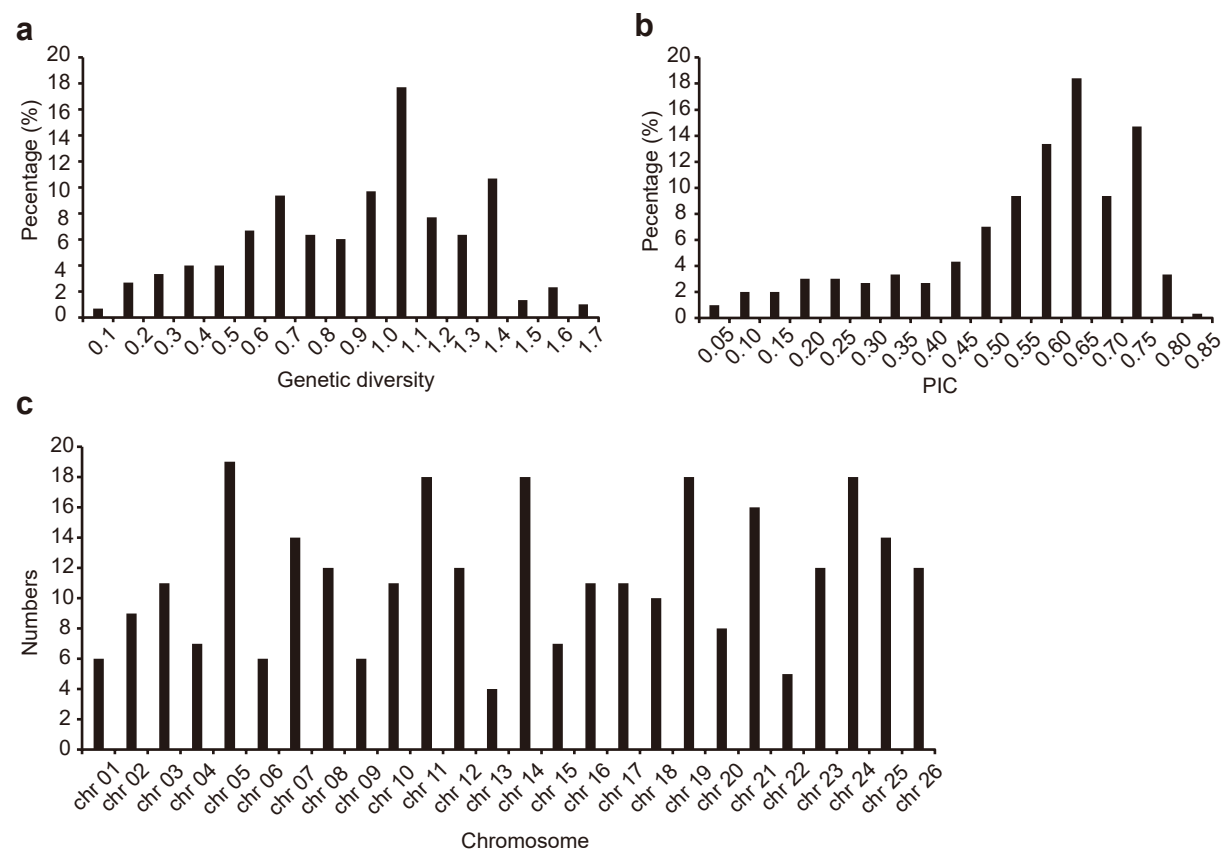

**Fig. S1**

Supplement: Supplementary file 4 — Figure S1. The diversity of 299 SSR markers. a Diversity index, b polymorphism information content, c distribution of markers in the upland cotton genome. (PDF 194 kb) [file 12870_2019_1725_MOESM4_ESM.pdf]

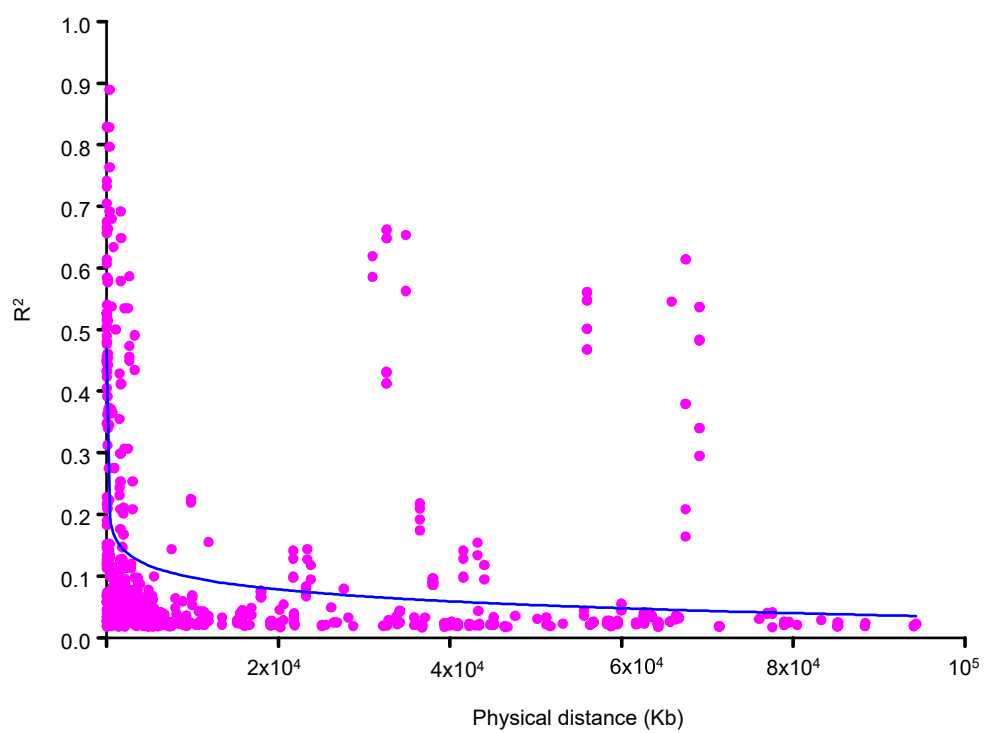

Fig. S2

Supplement: Supplementary file 6 — Figure S2. Linkage disequilibrium decay (R2). (PDF 222 kb) [file 12870_2019_1725_MOESM6_ESM.pdf]

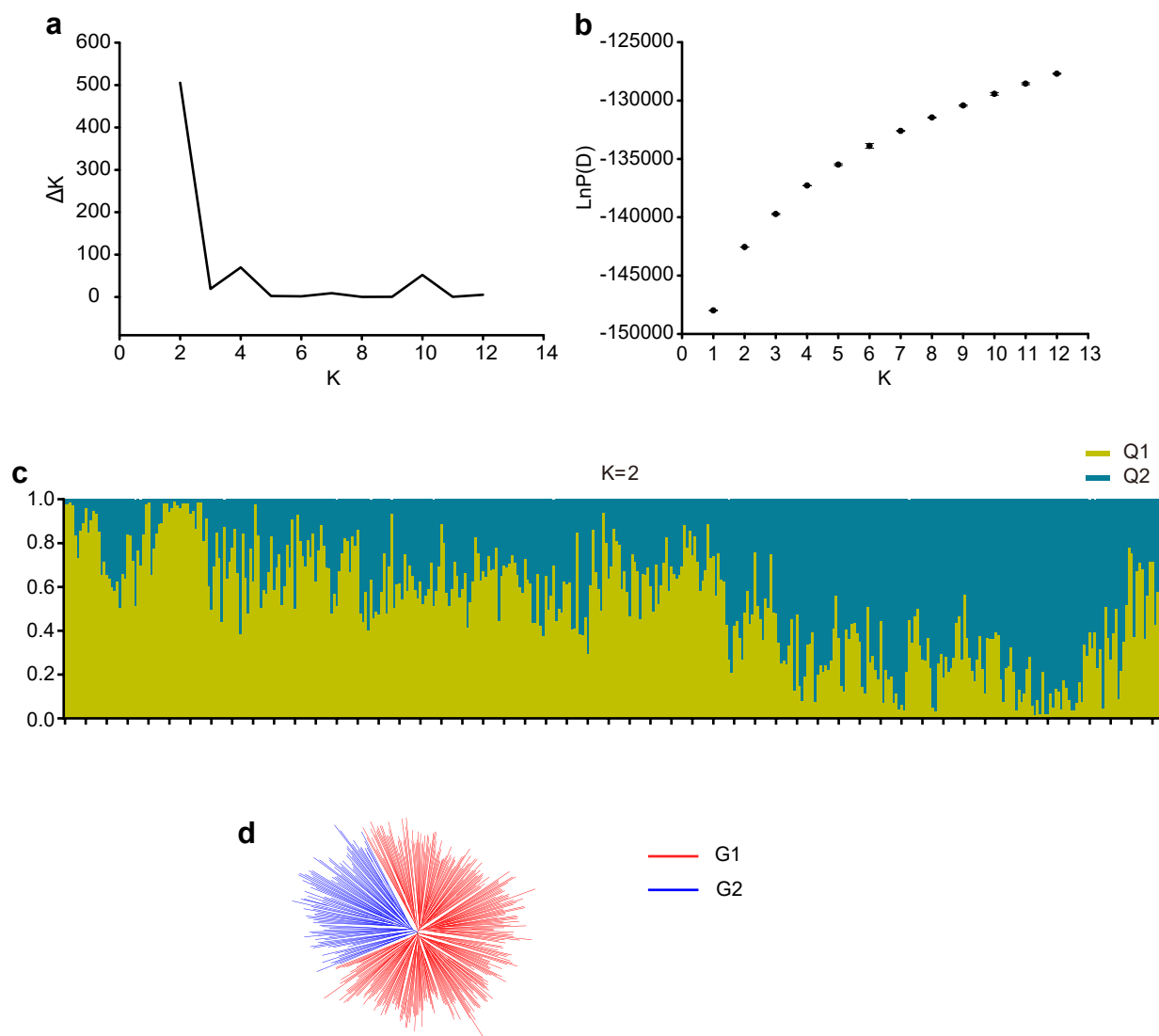

**Fig. S3**

Supplement: Supplementary file 7 — Figure S3. Population structure of the 419 accessions. a Mean LnP(D) values plotted from 1 to 12, b Ln(D) values plotted from 1 to 12, c Population structure of the 419 accessions based on STRUCTURE when K = 2, and d Neighbor-joining three of all accessions constructed from whole-genome SSRs. (PDF 433 kb) [file 12870_2019_1725_MOESM7_ESM.pdf]

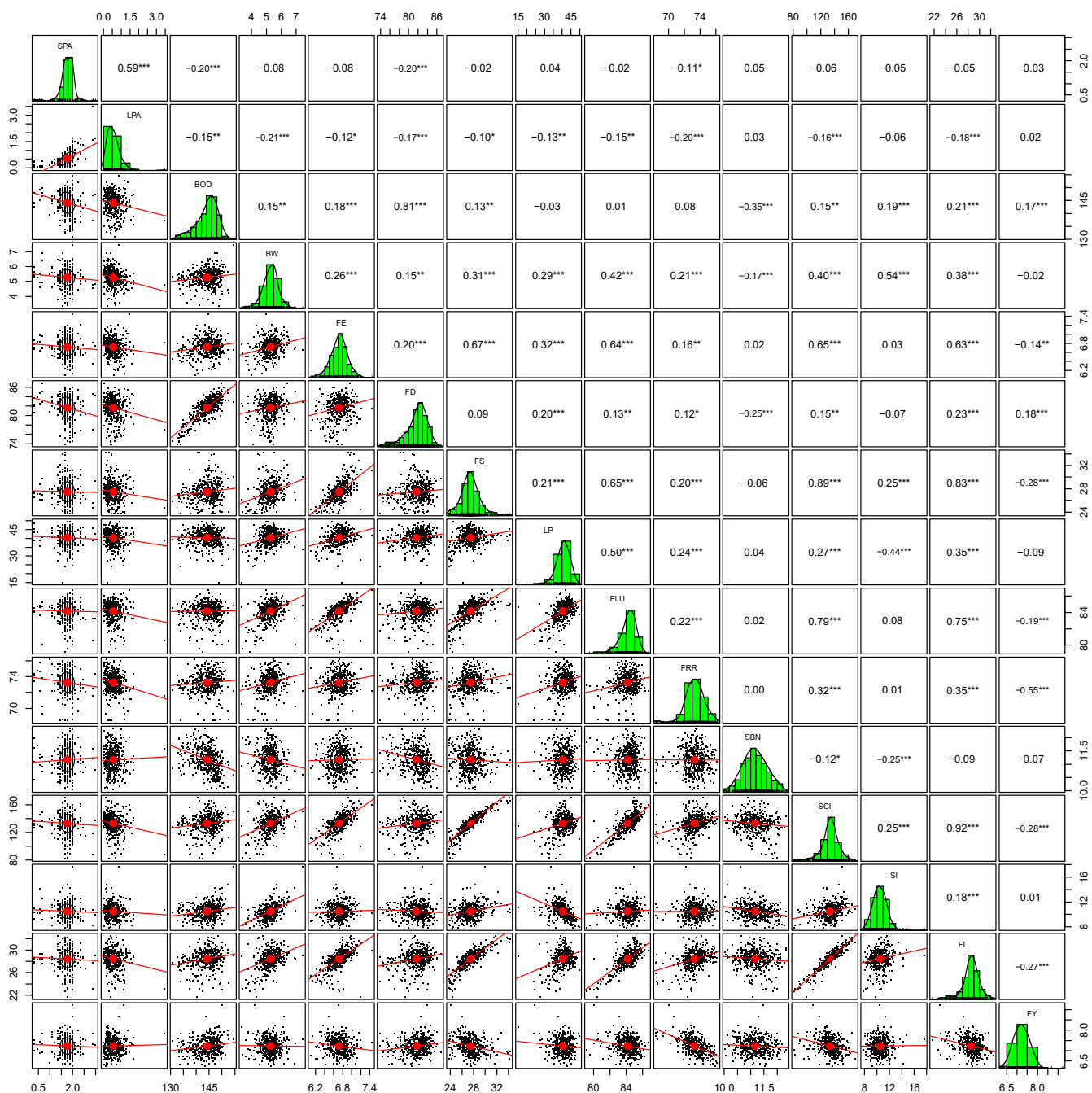

Fig. S4

Supplement: Supplementary file 9 — Figure S4. Frequency distribution of phenotypic variation of 15 agronomic traits and correlation coefficients among traits in 419 accessions. Abbreviations are defined as (SPA) stem pubescence amount, (LPA) leaf pubescence amount, (BOD) boll open date, (BW) weight per boll, (FE) fiber elongation, (FD) flowering date, (FS) fiber strength, (LP) lint percentage, (FLU) fiber length uniformity, (FRR) fiber reflectance rate, (SBN) sympodial brand number, (SCI) spinning consistency index, (SI) seed index, (FL) fiber upper half mean length, and (FY) fiber yellowness. (PDF 2459 kb) [file 12870_2019_1725_MOESM9_ESM.pdf]

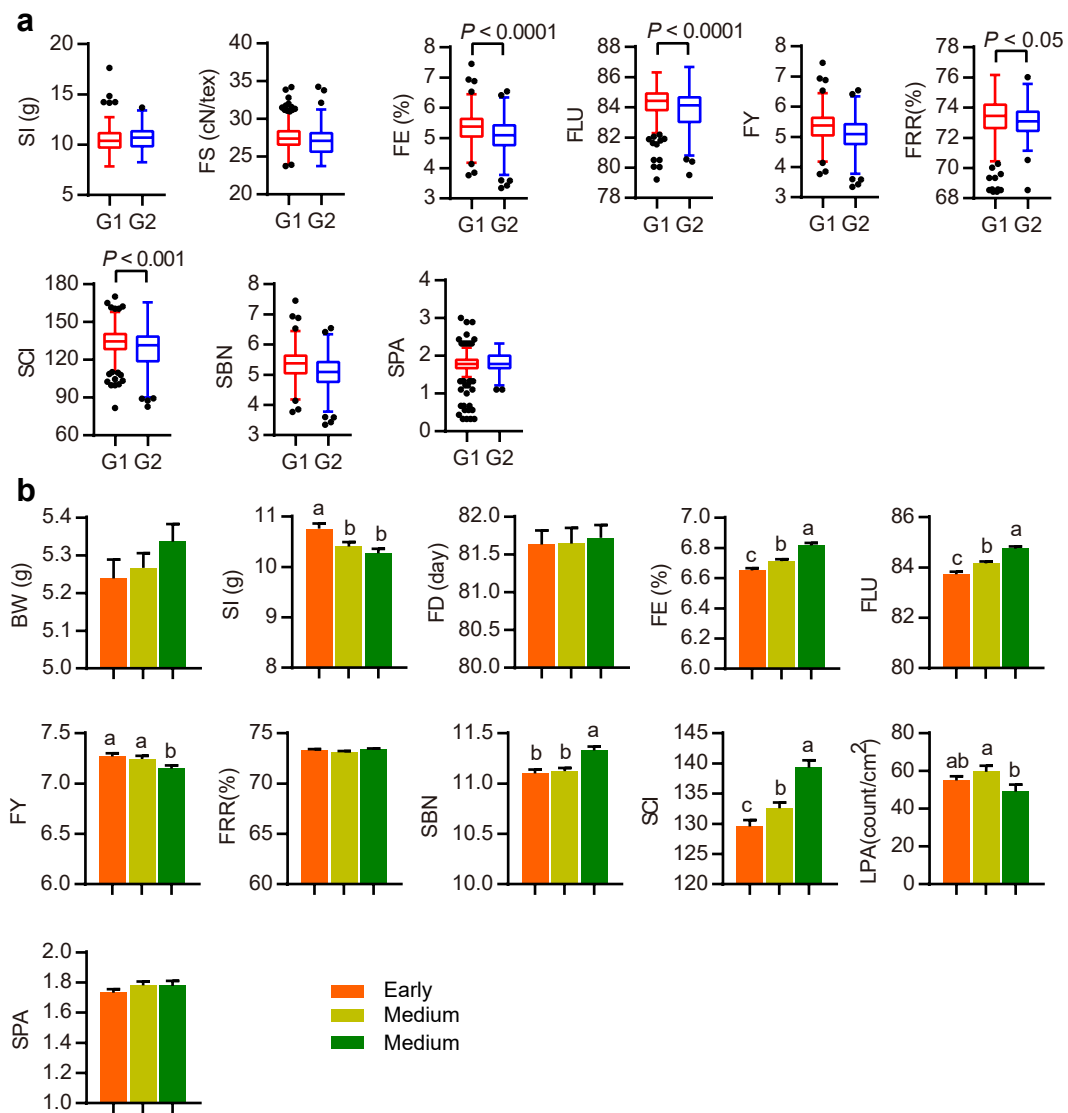

Fig. S5

Supplement: Supplementary file 10 — Figure S5. The variance analysis of phenotype traits in 2 groups and 3 breeding periods. a Comparison of investigated traits between 2 groups. b Comparison among 3 breeding periods. Horizontal lines in the boxplot represent the minimum, lower quartile, median, upper quartile and maximum, respectively, and black points represent mild outliers. P values in this and all other figures were derived within Duncan’s multiple comparison tests. Abbreviations are defined as (BW) weight per boll, (FD) flowering date, (FE) fiber elongation, (FS) fiber strength, (FLU) fiber length uniformity, (SBN) sympodial brand number, (FY) fiber yellowness, (LPA) leaf pubescence amount, (FRR) fiber reflectance rate, (SCI) spinning consistency index, (SI) seed index, and (SPA) stem pubescence amount. (PDF 316 kb) [file 12870_2019_1725_MOESM10_ESM.pdf]

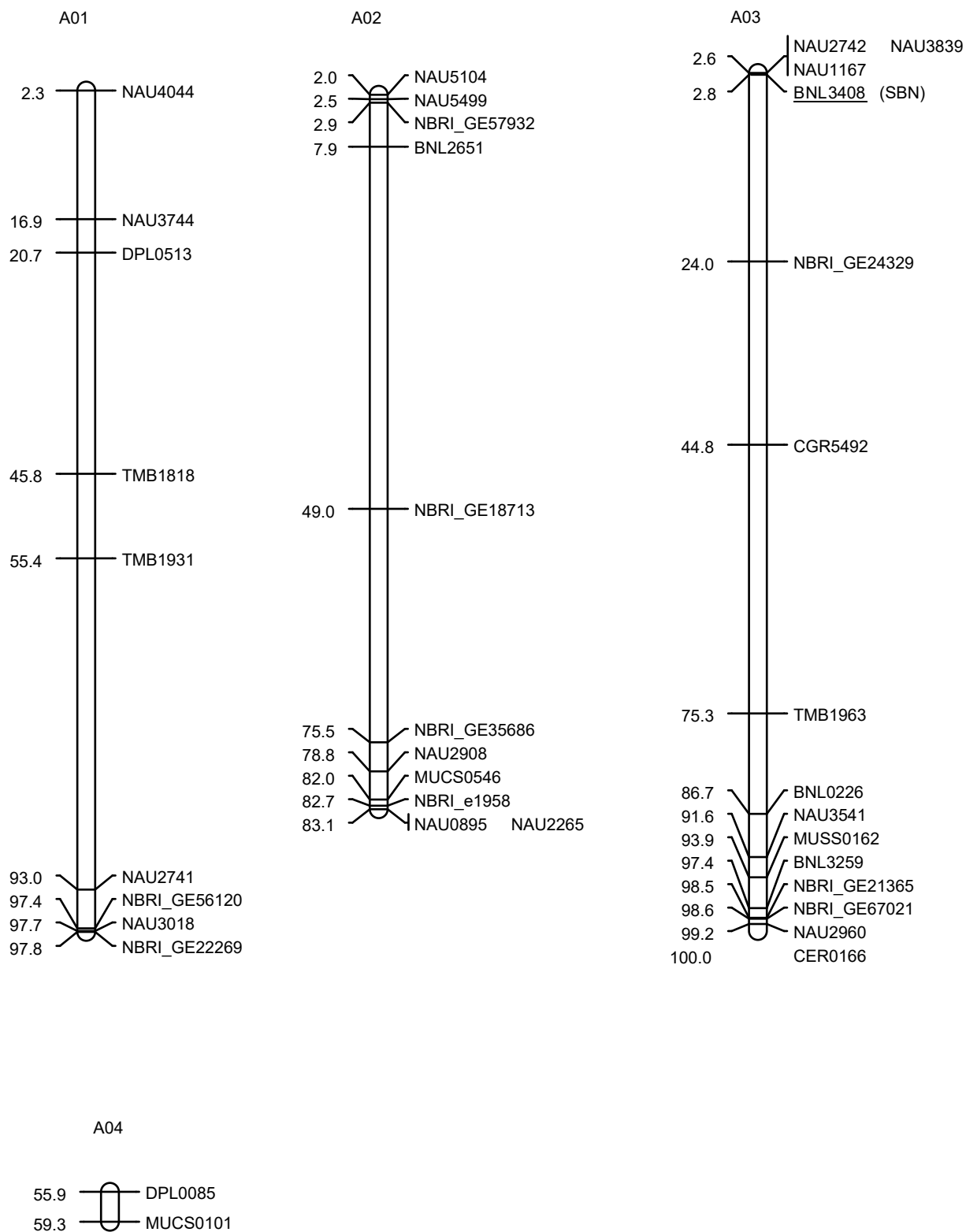

**Fig. S6**

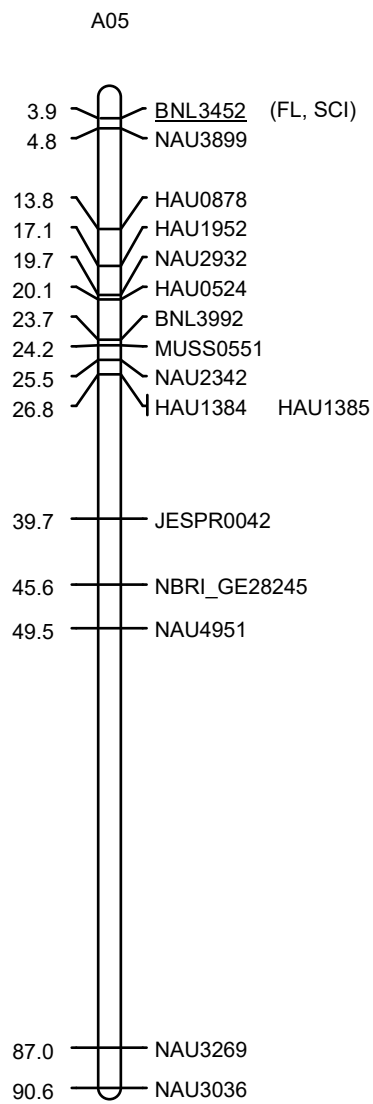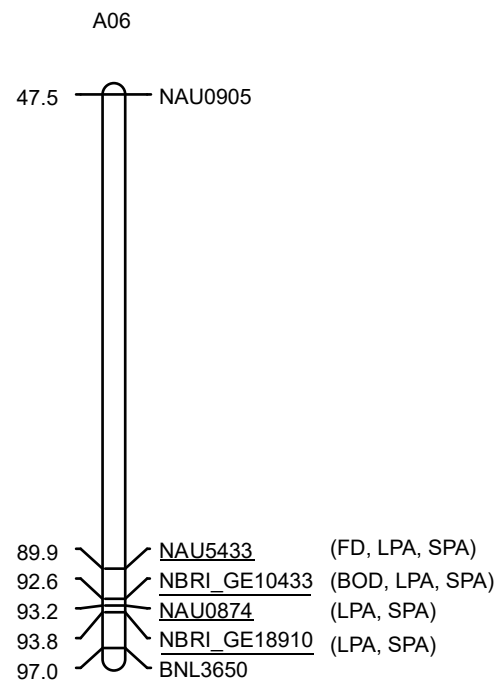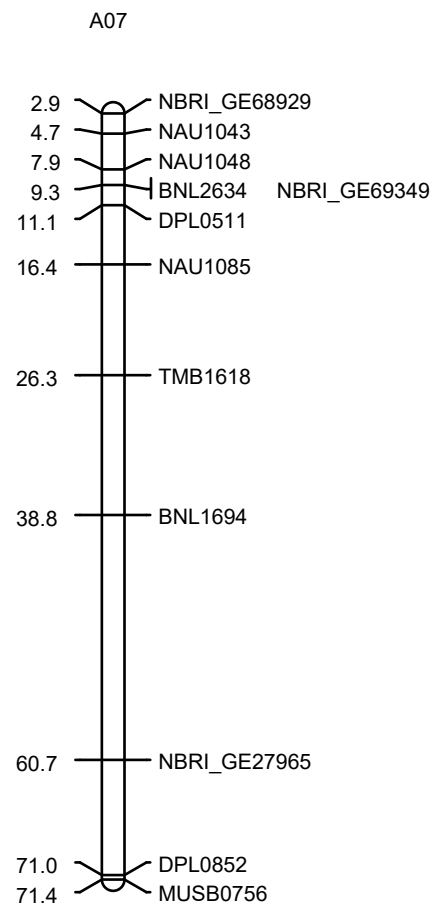

Fig. S6 continued

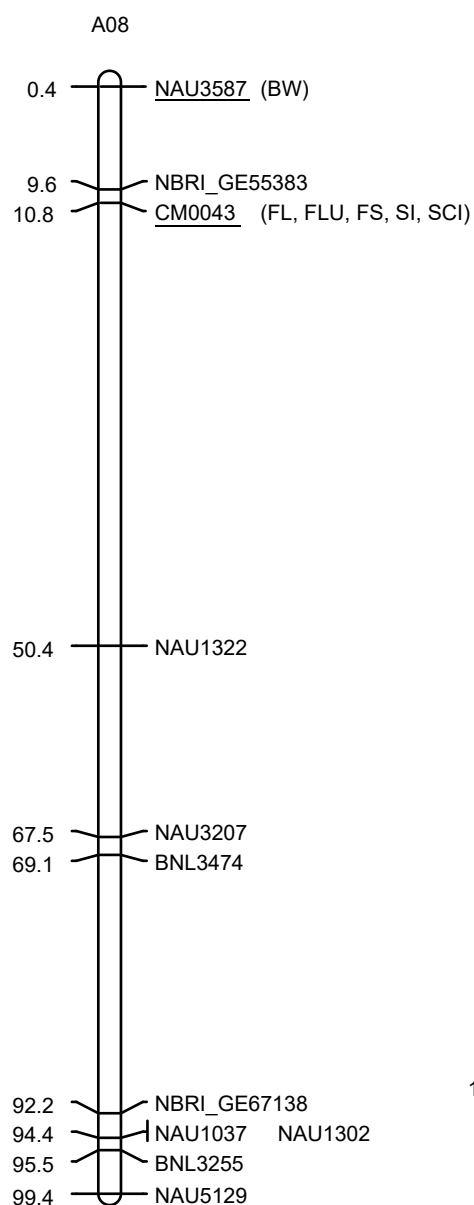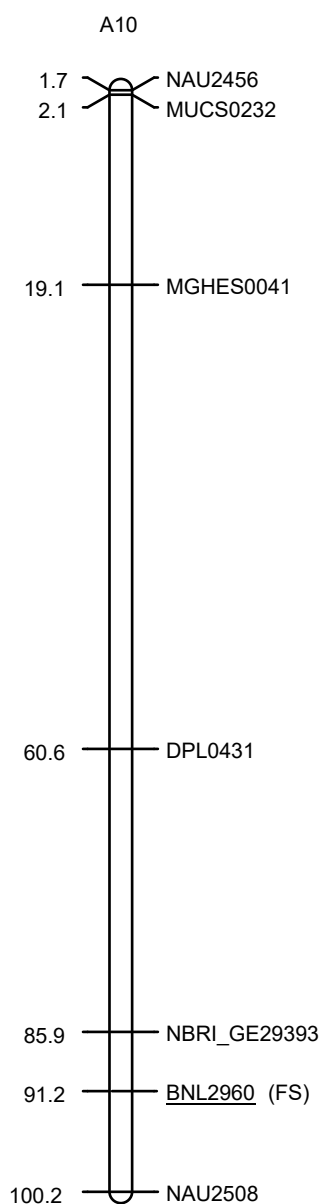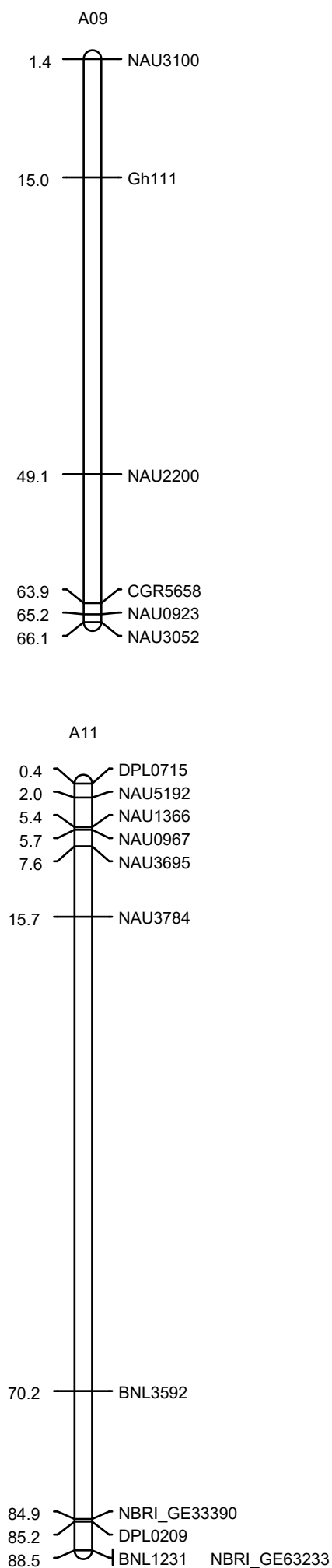

Fig. S6 continued

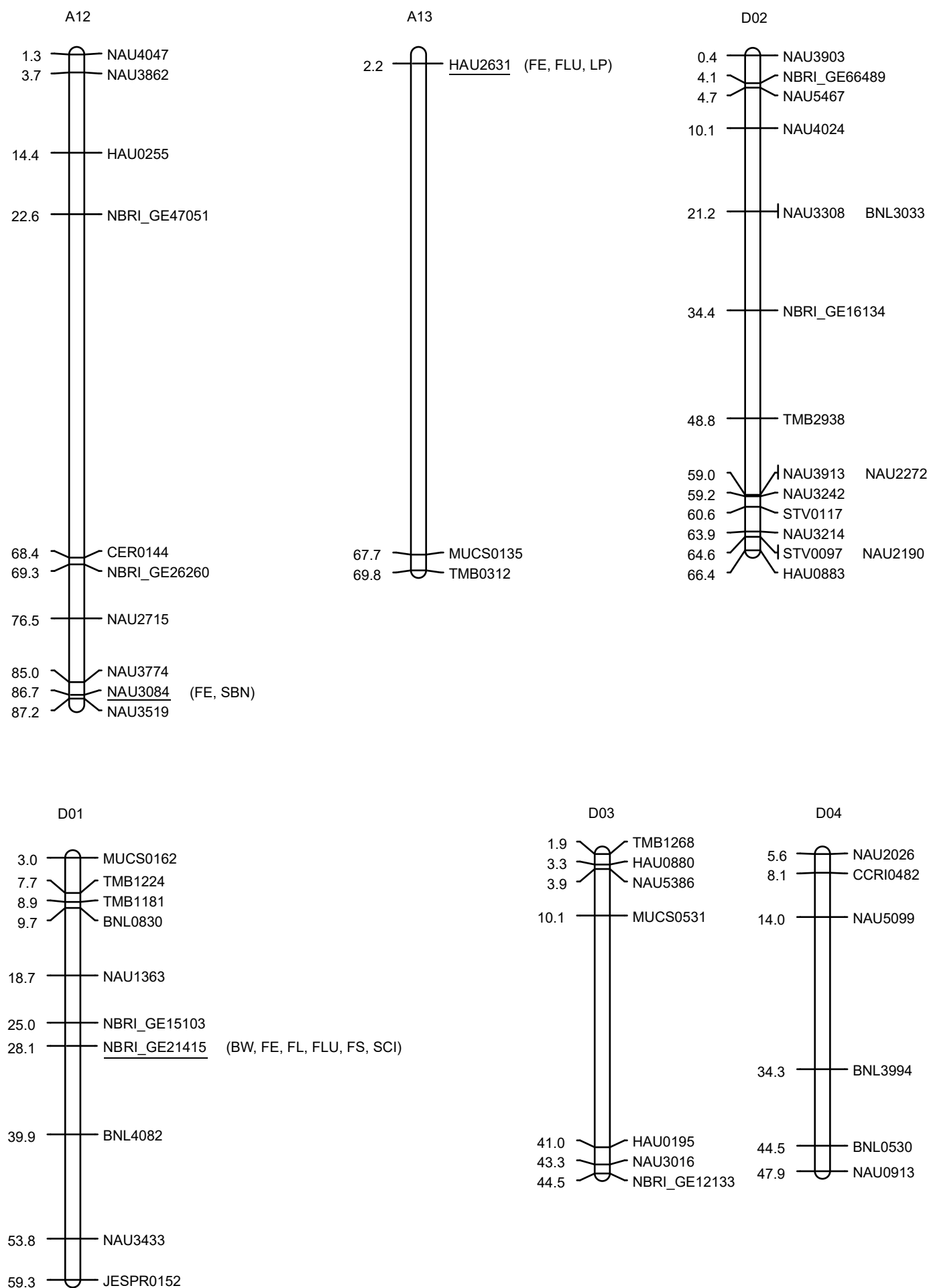

**Fig. S6** continued

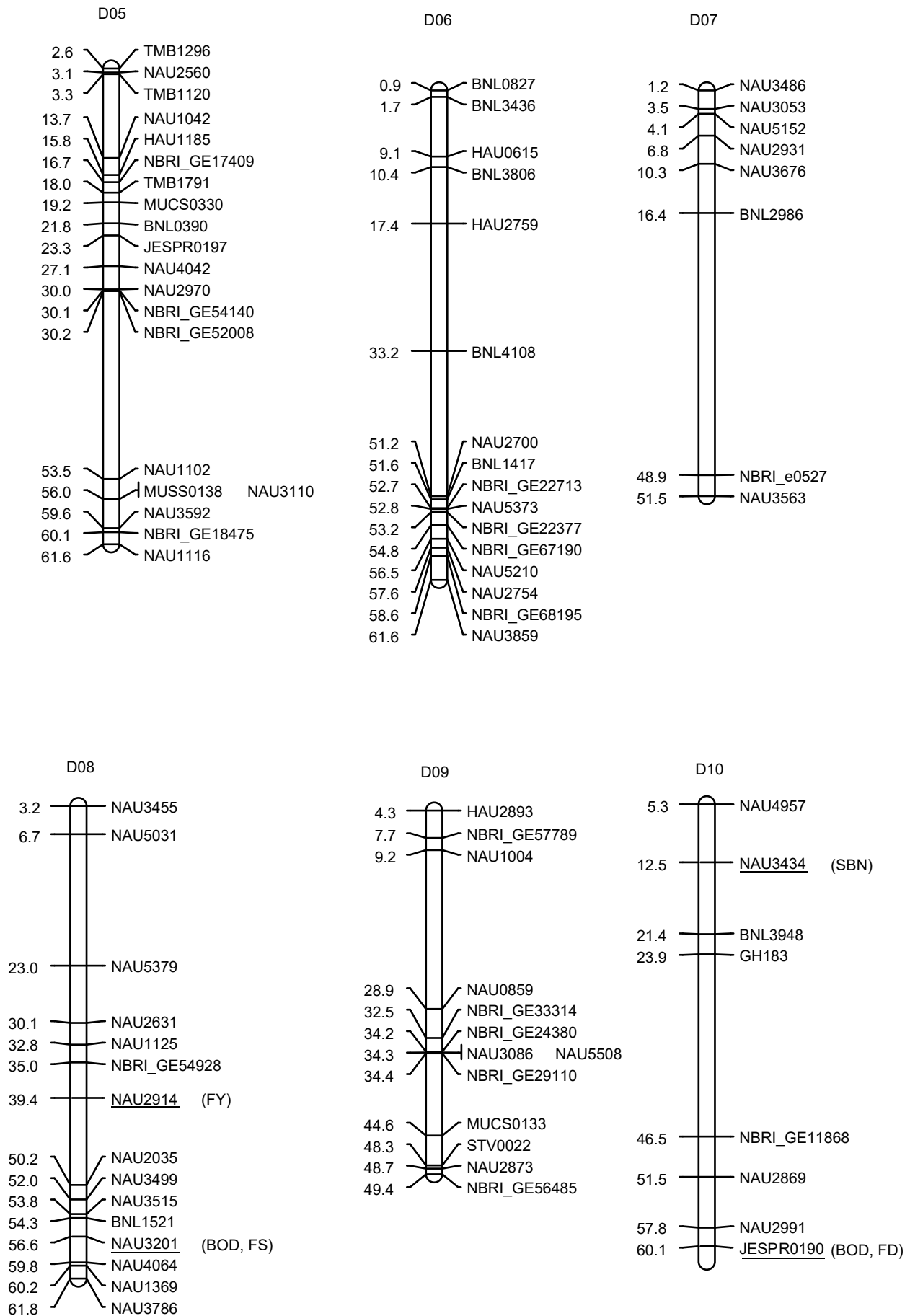

Fig. S6 continued

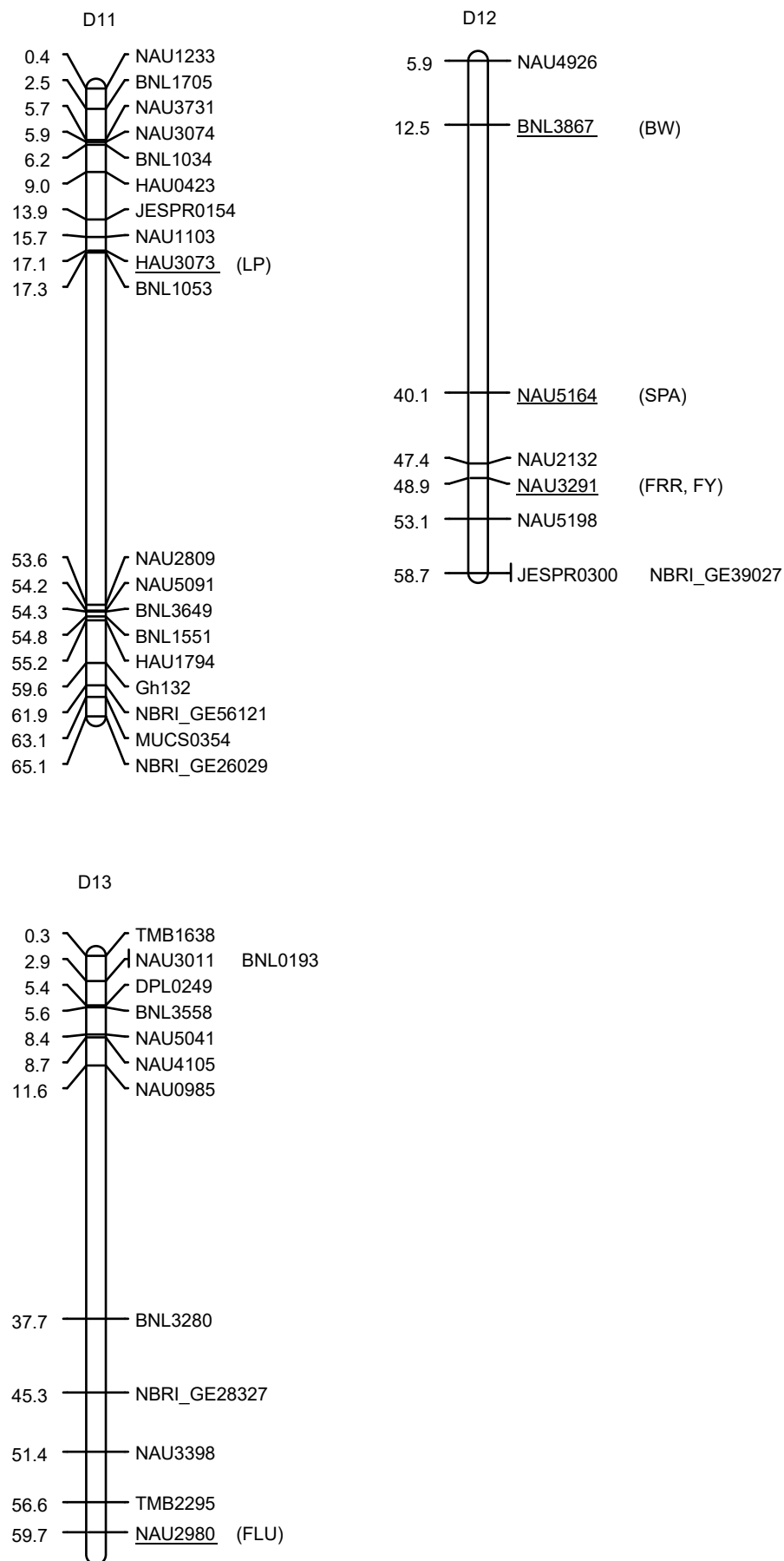

**Fig. S6** continued

Supplement: Supplementary file 11 — Figure S6. Physical map of 278 markers associated with 15 agronomic traits in 419 accessions. The loci with lines below the name were the markers significant associated (−log P > 4) with agronomic traits. Map distances were given in megabyte (Mb). The phenotypes of GWAS were within parentheses. Phenotypes were shown as stem pubescence amount (SPA), leaf pubescence amount (LPA), boll open date (BOD), weight per boll (BW), fiber elongation (FE), flowering date (FD), fiber strength (FS), lint percentage (LP), fiber length uniformity (FLU), fiber reflectance rate (FRR), sympodial brand number (SBN), spinning consistency index (SCI), seed index (SI), fiber upper half mean length (FL), and fiber yellowness (FY). (PDF 1224 kb) [file 12870_2019_1725_MOESM11_ESM.pdf]

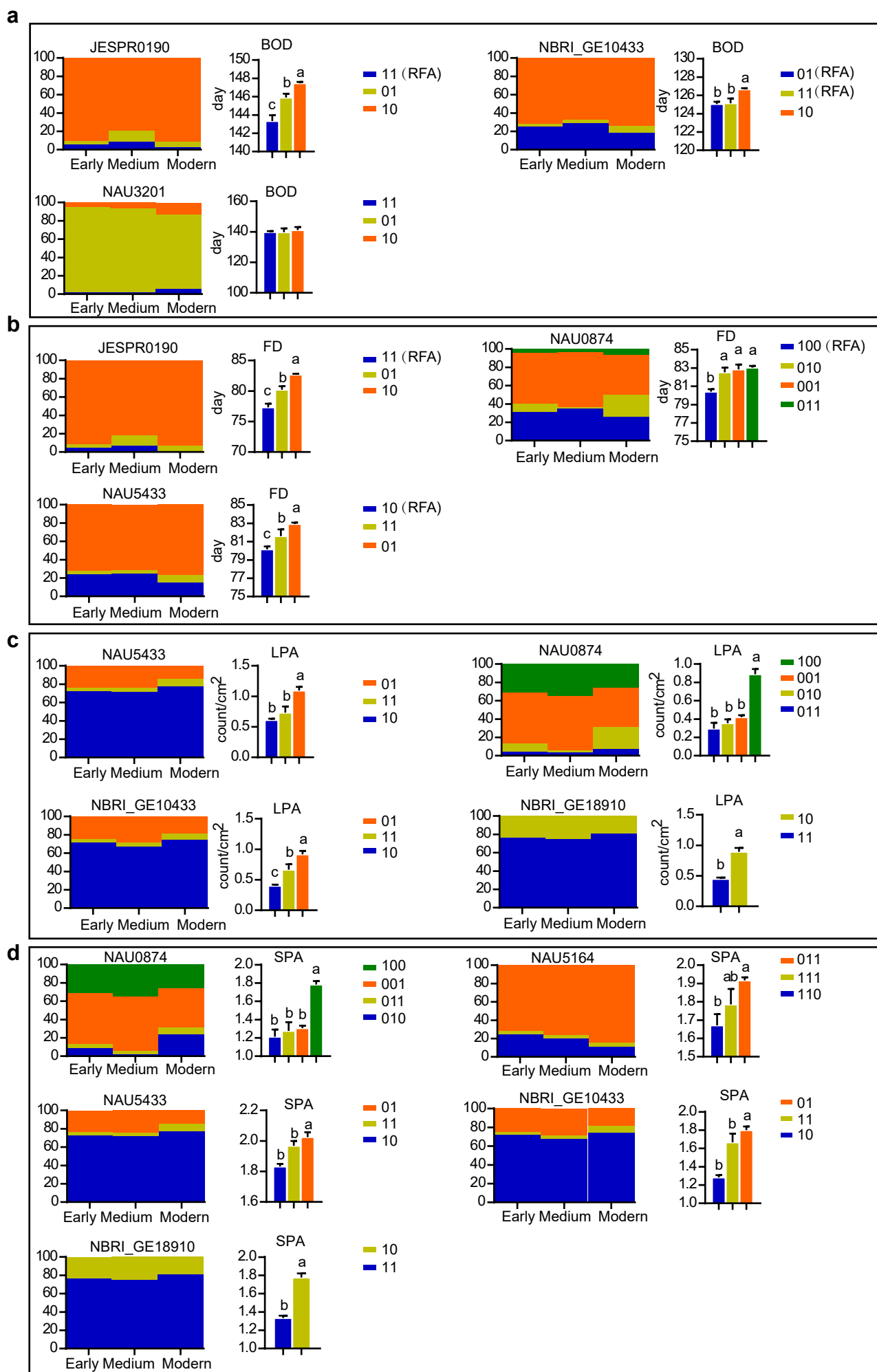

**Fig. S7**

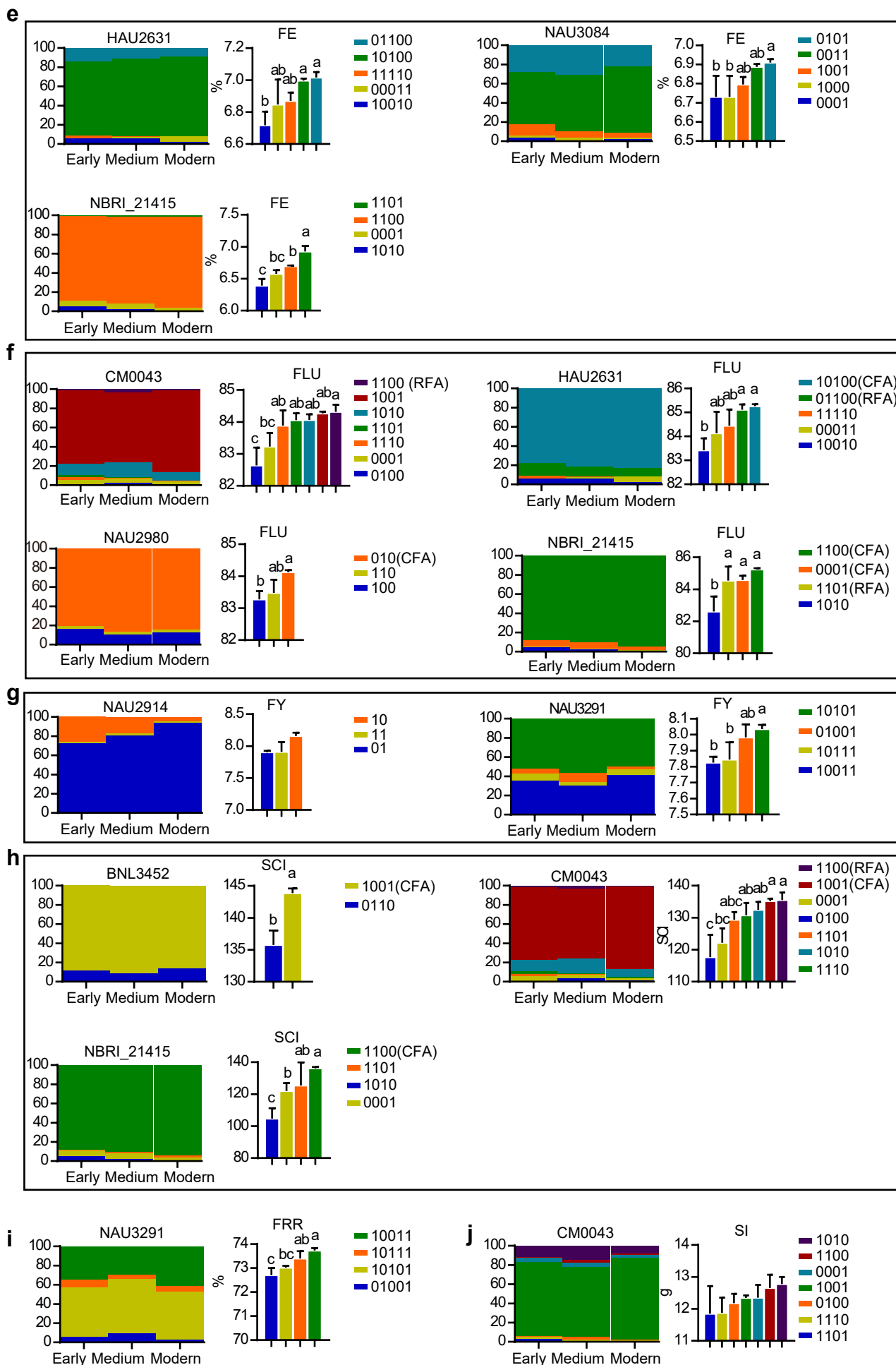

Fig. S7 continued

k

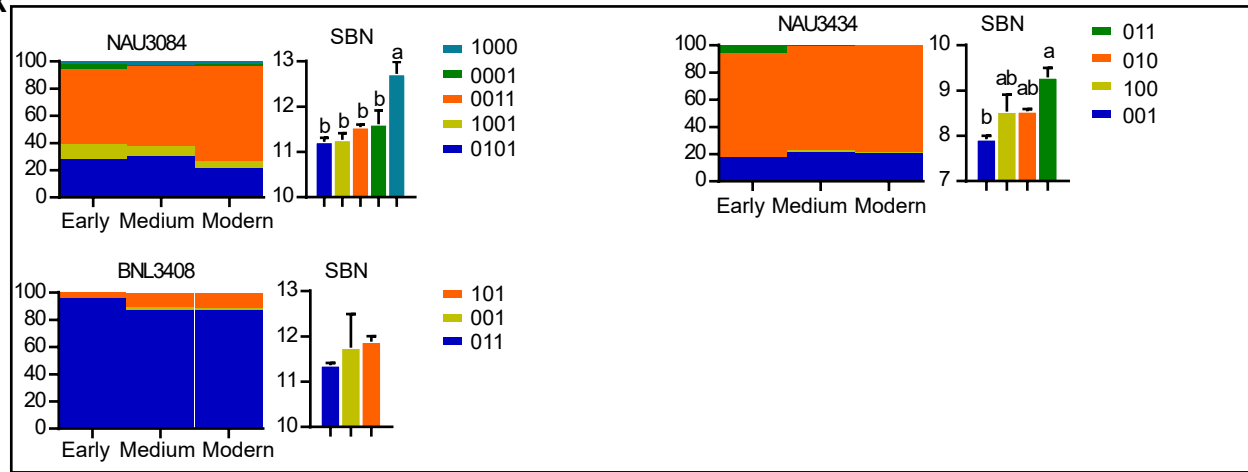

**Fig. S7** continued

Supplement: Supplementary file 12 — Figure S7. Allele distribution of 11 fiber-yield and quality traits and favorable alleles found in 419 upland cotton accessions early, medium, and modern varieties. a-k Frequency pile-up diagram for alleles distribution of 4 yield-fiber quality loci in 419 upland cotton accessions early, medium, and modern varieties (left). Histogram for 11 yield-fiber quality traits, based on the alleles of different loci, respectively (right). The significance (P < 0.05) was tested by Duncan’s multiple comparison tests. We also identified rare favorable alleles (RFAs) as those that were the frequency of FAs < 25% and common favorable alleles (CFAs) as the frequency of favorable alleles > 70%, respectively. a-k Different traits in early, medium, and modern periods, a (BOD) boll open date, b (FD) flowering date, c (LPA) leaf pubescence amount, d (SPA) stem pubescence amount, e (FE) fiber elongation, f (FLU) fiber length uniformity, g (FY) fiber yellowness, h (SCI) spinning consistency index, i (FRR) fiber reflectance rate, j (SI) seed index, and k (SBN) sympodial brand number. (PDF 724 kb) [file 12870_2019_1725_MOESM12_ESM.pdf]
